# Eosinophil Dynamics as Prognostic Indicators in Immunotherapy for Pulmonary Lymphoepithelioma-Like Carcinoma

**DOI:** 10.7759/cureus.94131

**Published:** 2025-10-08

**Authors:** Chun Yee Ryan Ho, Ka Man Cheung, Chung Hang James Chow, Gavin Tin Chun Cheung

**Affiliations:** 1 Department of Clinical Oncology, Queen Elizabeth Hospital, Kowloon, HKG; 2 Department of Clinical Oncology, The Chinese University of Hong Kong, Shatin, HKG; 3 Department of Oncology, United Christian Hospital, Kowloon, HKG

**Keywords:** eosinophil, immunotherapy, prognostic biomarker, progression-free survival, pulmonary lymphoepithelioma-like carcinoma

## Abstract

Introduction: Pulmonary lymphoepithelioma-like carcinoma (pLELC) is a rare form of non-small cell lung cancer. As immunotherapy (IO) becomes an important treatment for advanced pLELC, identifying reliable prognostic markers is crucial for guiding clinical decisions and resource allocation.

Methods: This multicentre retrospective cohort study included 26 patients with advanced or metastatic pLELC who received palliative IO at two tertiary hospitals in Hong Kong from 2010 to 2023. Clinical and haematological data, particularly eosinophil peak timing, eosinophil-to-neutrophil ratio (ENR), and neutrophil-to-lymphocyte ratio (NLR), were obtained at each treatment cycle. Progression-free survival (PFS) and overall survival (OS) were evaluated using Kaplan-Meier analysis. Cox regression models, complemented by landmark and sensitivity analyses, were used to determine independent prognostic factors.

Results: Early eosinophil peak (≤5 weeks after starting IO) was significantly linked to shorter PFS (HR 7.0, p = 0.007), while ENR <0.054 independently predicted poorer PFS (HR 2.9, p = 0.04). Neither ENR nor NLR showed significant associations with OS in multivariate analysis. Delayed eosinophil peaks were associated with more favourable PFS, supporting the prognostic value of eosinophil kinetics. Sensitivity analyses confirmed these findings were robust across patient subgroups.

Conclusions: Eosinophil peak timing is a practical, independent biomarker for identifying pLELC patients less likely to benefit from IO and may enhance patient prognostication and management. Further prospective studies in larger, multi-centre cohorts are needed to validate the clinical use of eosinophil dynamics in IO monitoring for pLELC.

## Introduction

Pulmonary lymphoepithelioma-like carcinoma (pLELC) is an uncommon subtype of non-small cell lung cancer (NSCLC) [1] associated with Epstein-Barr virus (EBV) infection [2], predominantly affecting young non-smokers in Southeast Asia [3,4]. pLELC has a distinctive genomic profile that differentiates it from primary NSCLC subtypes but is similar to that of EBV-associated nasopharyngeal carcinoma (NPC) [5].

While platinum-based chemotherapy has remained the current mainstay approach for pLELC [6], the optimal treatment for advanced pLELC is uncertain. Unlike NSCLC, pLELC rarely has actionable mutations such as epidermal growth factor receptor (EGFR) or anaplastic lymphoma kinase (ALK) [5]. Despite high PD-L1 expression [7-9], immunotherapy (IO) ’s role in pLELC is limited due to under-representation in clinical trials. Although IO shows efficacy in advanced lung cancer [10-19], its use in pLELC remains less proven. Recent studies indicate IO's potential efficacy [20,21], with first-line chemoimmunotherapy (CIO) extending progression-free survival (PFS) more than traditional chemotherapy [20,22].

Recent studies have suggested that peripheral blood leukocytes have prognostic value in NSCLC [23-25]. A study by Tanizaki et al. indicated that patients with a low baseline absolute neutrophil count, high eosinophil and lymphocyte counts, experienced improved survival outcomes when treated with IO [23]. Another study demonstrated that a higher peak eosinophil-to-neutrophil ratio (ENR) was associated with improved response and survival in metastatic NSCLC treated with IO, whereas the neutrophil-to-lymphocyte ratio (NLR) did not significantly predict these outcomes [26]. However, there is no available data on eosinophil dynamics in pLELC. A nomogram has recently been developed using a machine learning approach for prognostic assessment in patients with pLELC undergoing IO [27]. This model predicts PFS and response using clinical and haematological parameters, including eosinophil counts and NLR. However, due to the black-box nature of AI models, the contribution of each factor to the predictions remains unclear. Therefore, this study aims to investigate the potential predictive value of eosinophil dynamics and validate the prognostic value of the ENR and NLR in pLELC.

## Materials and methods

Study design and setting

This multicenter retrospective cohort study was conducted at two tertiary care hospitals in Hong Kong, Queen Elizabeth Hospital and United Christian Hospital, Kowloon, Hong Kong.

Participants

The inclusion criteria encompassed all consecutive advanced or metastatic stage patients diagnosed with pLELC, planning to receive systemic treatment, who had undergone IO with or without concurrent chemotherapy. Patients who received adjuvant immunotherapy as part of their definitive treatment, as well as those with unclear records of prior treatment, were excluded.

Data sources and collection

Patients with metastatic pLELC from 2010 to 2023 were identified in the cancer registry. Demographics, clinical characteristics, and outcomes were systematically collected and analysed. Blood tests were done before each IO cycle to monitor haematological parameters. For each patient, the eosinophil peak was defined as the first observed maximum value following treatment initiation, and the corresponding ENR and NLR values were recorded at this specific time point. Clinical data were described using medians, interquartile ranges, and percentages.

Bias and sensitivity analyses

To minimise immortal time bias, a landmark analysis was conducted by excluding patients who either survived less than five weeks after starting IO (n=2), and patients treated with concurrent IO and platinum chemotherapy were excluded (n=2) to account for their potential impact on neutrophil counts. The five-week cutoff was chosen to reflect the median time to eosinophil peak in the dataset. Sensitivity analyses assessed the robustness of results by repeating analyses in the full cohort to better control for confounding and bias.

Molecular analysis

For molecular analysis, gene sequencing via multiplex polymerase chain reaction (PCR) targeted EGFR hotspot mutations. ALK and proto-oncogene 1 receptor tyrosine kinase (ROS-1) translocations were identified using immunohistochemistry (IHC). PD-L1 tumour proportion score (TPS) was assessed using IHC with the PharmDx 22C3 antibody.

Statistical analysis

PFS and overall survival (OS) were estimated using the Kaplan-Meier method. Univariate and multivariate Cox proportional hazards models identified prognostic factors, with a 5% significance level. To account for multiple testing in the univariate analysis of the landmark cohort, p-values were adjusted using the Benjamini-Hochberg false discovery rate (FDR) correction. Significant factors from univariate analysis were prioritised in multivariate analysis. To minimise the risk of overfitting given the small sample size in the landmark cohort (n=22), only the two most clinically and statistically significant predictors from univariate analyses - timing of peak eosinophil count and ENR - were included in the multivariate Cox model for PFS. Data analysis was conducted using RStudio (version 2024.04.2; RStudio Team, Boston, MA) and the survival package (v3.7-0).

## Results

In a database of 200 pLELC patients, 30 received IO, with 26 eligible cases analysed after excluding four for adjuvant phase IO. Most were female (n=20) with a median age of 63, an interquartile range (IQR) of 56-74 years, and 80.3% were non-smokers. Additionally, 73% had a performance status of 0-1. Prior treatments included platinum-based chemotherapy (69%), gemcitabine (77%), and capecitabine (50%). With similar PD-L1 TPS (≥ 50% vs < 50%) across groups and no EGFR, ALK, or ROS-1 mutations found, all IO treatments were given for metastatic disease - eight as first-line treatment (1L) (pembrolizumab = 7, atezolizumab = 1) and 18 as second-line or subsequent treatment (2L) (nivolumab = 11, atezolizumab = 6, pembrolizumab = 1) (Table 1).

**Table 1:** Baseline demographics of the full cohort (n=26) This table summarises the baseline demographic and clinical characteristics of the 26 patients with metastatic pulmonary lymphoepithelioma-like carcinoma (pLELC) included in this study. Most were female (n=20) with a median age of 65, and 80.3% were non-smokers. Additionally, 73% had a performance status of 0-1. Prior treatments included platinum-based chemotherapy (69%), gemcitabine (77%), and capecitabine (50%). With similar PD-L1 TPS (≥ 50% vs < 50%) across groups and no EGFR, ALK, or ROS-1 mutations found, all IO treatments were given for metastatic disease - eight as first-line treatment (1L) (pembrolizumab = 7, atezolizumab = 1) and 18 as second-line or subsequent treatment (2L) (nivolumab = 11, atezolizumab = 6, pembrolizumab = 1) 1L: first-line treatment; 2L: second-line or subsequent treatment; ECOG PS: Eastern Cooperative Oncology Group performance status; PD-L1: programmed cell death ligand 1; TPS: tumour proportion score; EGFR: epidermal growth factor receptor; ALK: anaplastic lymphoma kinase; ROS-1: ROS proto-oncogene 1 receptor tyrosine kinase; SBRT: stereotactic body radiation therapy

Variables		Value
Age	Median age (years)	65
Sex	Male (n, %)	6 (23%)
	Female (n, %)	20 (77%)
Smoking status	Smoker (n, %)	5 (19%)
	Non-smoker (n, %)	21 (81%)
ECOG PS	0-1	19 (73%)
	2	7 (27%)
	3 or above	0 (0%)
Metastatic sites	Lung	12 (46%)
	Pleura	11 (42%)
	Intra-thoracic lymph nodes	20 (77%)
	Extra-thoracic lymph nodes	6 (23%)
	Liver	6 (23%)
	Bone	5 (19%)
	Pleural effusion	3 (12%)
	Adrenal	1 (4%)
	Pericardial effusion	2 (8%)
	Peritoneum	1 (4%)
PD-L1 (TPS)	50% or above (n, %)	10 (38%)
	<50% (n, %)	10 (38%)
	Unknown (n, %)	6 (23%)
EGFR status	Positive (n,%)	0 (0%)
	Negative (n, %)	19 (73%)
	Unknown (n, %)	7 (27%)
ALK status	Positive (n, %)	0 (0%)
	Negative (n, %)	17 (65%)
	Unknown (n, %)	9 (35%)
ROS-1 status	Positive (n, %)	0 (0%)
	Negative (n, %)	15 (58%)
	Unknown (n, %)	11 (42%)
Lines of treatment	1L (n, %)	8 (31%)
	2L (n, %)	18 (69%)
Prior systemic treatment	Platinum-based chemotherapy	18 (69%)
	Capecitabine	13 (50%)
	Gemcitabine	20 (77%)
	Taxane	5 (19.2%)
Prior local treatment	Surgical resection	8 (31%)
	Chemoradiotherapy	6 (23%)
	SBRT	1 (4%)
Immune-checkpoint inhibitor	Pembrolizumab	8 (31%)
	Atezolizumab	7 (27%)
	Nivolumab	11 (42%)

**Figure 1:**
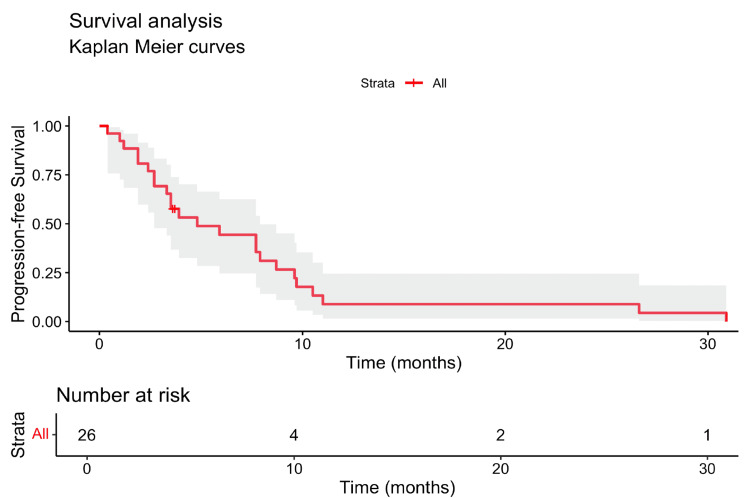
Kaplan-Meier curve showing progression-free survival (PFS) in the full cohort The median PFS was 3.8 months (95% confidence interval: 0.4-30.9 months). PFS: progression-free survival

**Figure 2:**
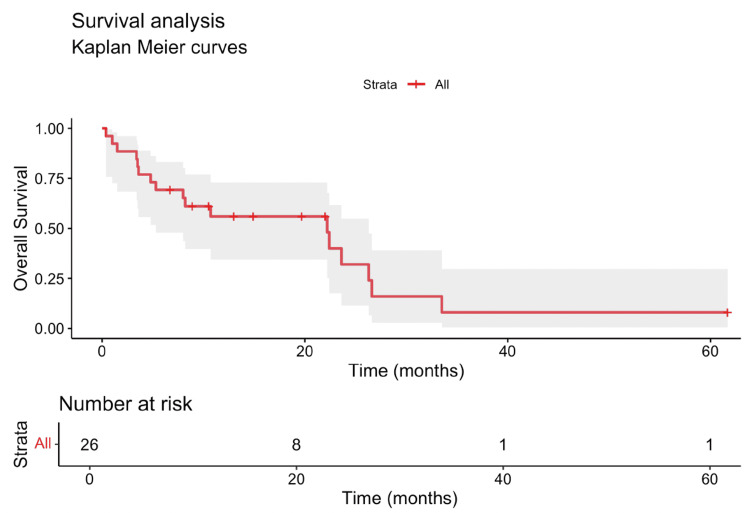
Kaplan-Meier curve showing the overall survival (OS) in the full cohort The median OS was 10.5 months (95% CI: 0.4-61.7 months). OS: overall survival

**Figure 3:**
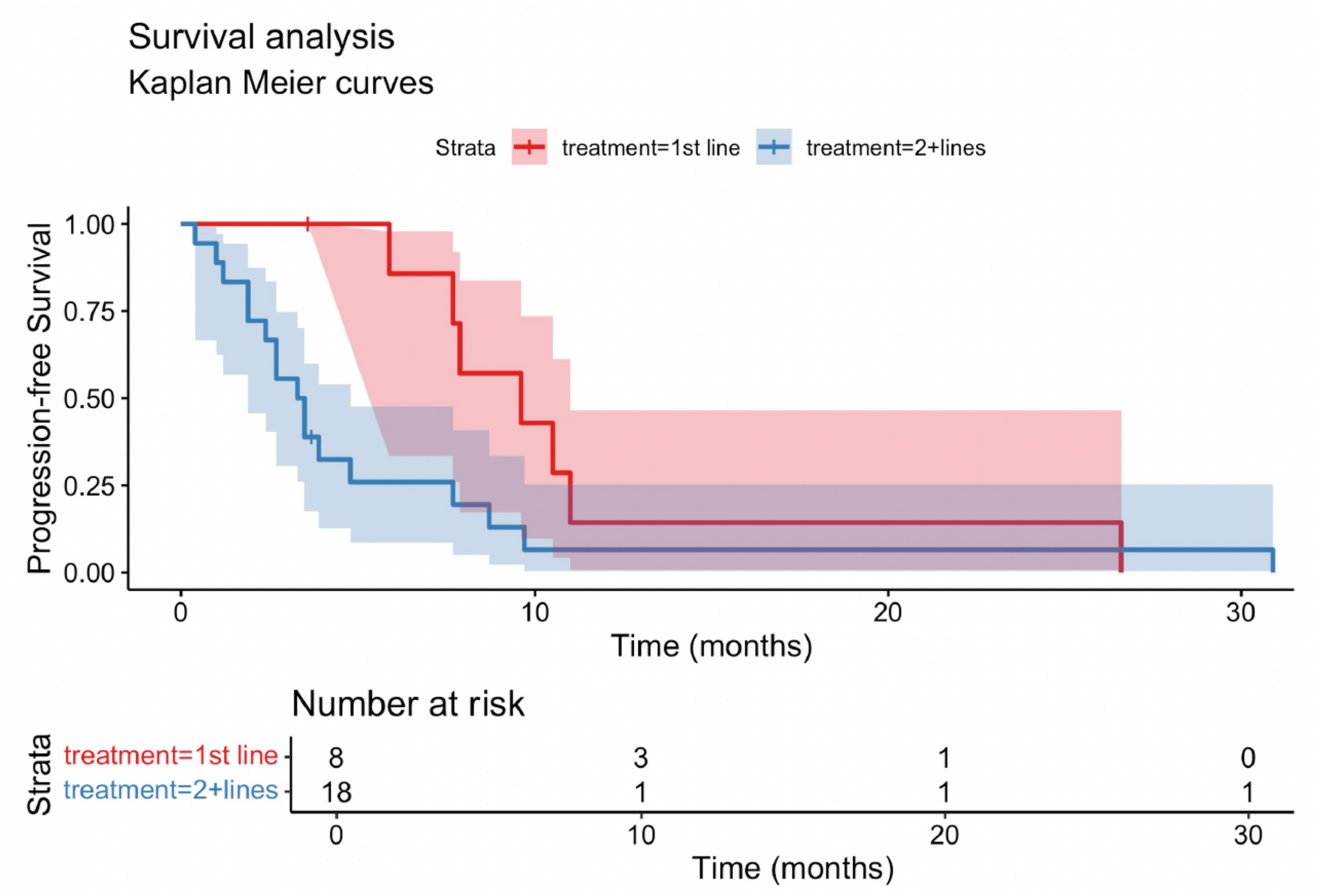
Kaplan-Meier curve of progression-free survival (PFS) by line of treatment This figure presents Kaplan-Meier estimates of progression-free survival (PFS) for patients with metastatic pulmonary lymphoepithelioma-like carcinoma (pLELC) undergoing immunotherapy, grouped by treatment line. The PFS for first-line (1st line) treatment shows a median of 9.6 months, while those receiving second-line or beyond (2+ lines) treatment have a median PFS of 3.4 months. PFS: progression-free survival; 1st line: first-line treatment; 2+ lines: second-line or beyond treatment

**Figure 4:**
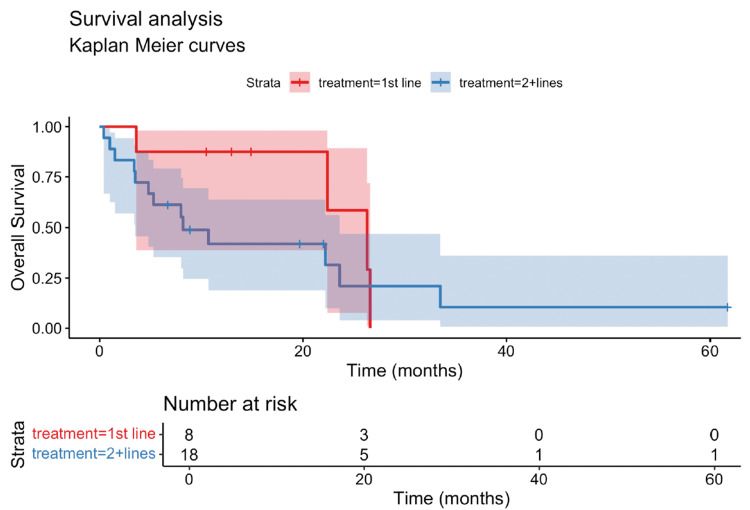
Kaplan-Meier curve of the overall survival (OS) by line of treatment This figure presents the Kaplan-Meier estimates of the overall survival (OS) for patients with metastatic pulmonary lymphoepithelioma-like carcinoma (pLELC) undergoing immunotherapy, grouped by treatment line. The OS for first-line (1st line) treatment shows a median of 26.3 months, while those receiving second-line or beyond (2+ lines) treatment have a median OS of 8.2 months. OS: overall survival; 1st line: first-line treatment; 2+ lines: second-line or beyond treatment

The overall response rate (ORR), defined as the proportion of patients achieving a complete response or partial response by RECIST v1.1 criteria, was 15.4% in our cohort. The median PFS was 3.8 months (95% confidence interval (CI): 0.4-30.9 months) (Figure 1), while the median OS was 10.5 months (95% CI: 0.4-61.7 months) (Figure 2). For 1L treatment, the median PFS was 9.6 months (95% CI: 5.9-11 months) (Figure 3), and the median OS was 26.3 months (95% CI: 3.6 to not reached) (Figure 4). In 2L, the PFS was 3.4 months (95% CI: 1.9-4.8 months) (Figure 3) and OS was 8.2 months (95% CI: 3.5-23.6 months) (Figure 4). Only one grade 3 or higher side effect, dermatitis (3%), was recorded.

**Figure 5:**
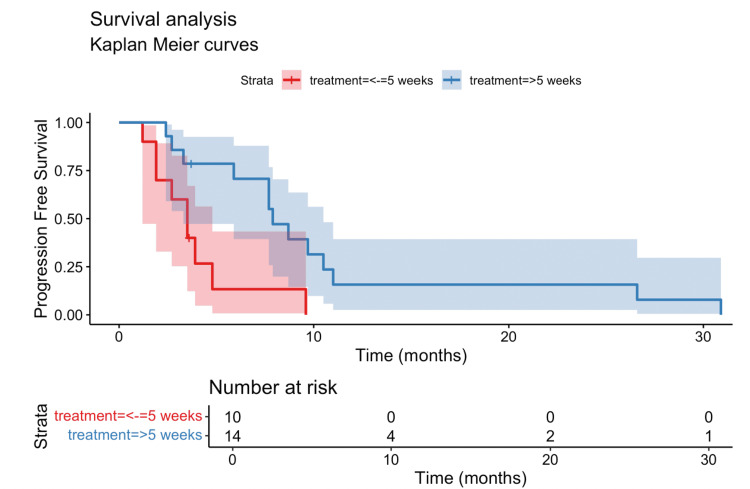
Kaplan-Meier curve of the progression-free survival (PFS) stratified by the time to peak eosinophil count (≤5 weeks vs >5 weeks) Patients with an early peak eosinophil count (≤5 weeks) had significantly shorter median PFS compared to those with a delayed peak (>5 weeks) (3.5 vs 7.9 months). PFS: progression-free survival

**Figure 6:**
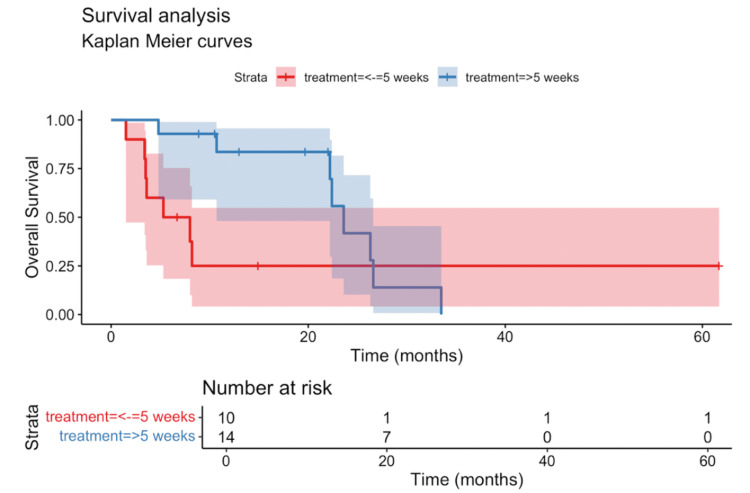
Kaplan-Meier curve of the overall survival (OS) stratified by the time to peak eosinophil count (≤5 weeks vs >5 weeks) Patients with an early peak eosinophil count (≤5 weeks) had significantly shorter median OS compared to those with a delayed peak (>5 weeks) (6.7 vs 23.6 months). OS: overall survival

Our study examined haematological markers' impact on PFS and OS. While clinical parameters did not reach statistical significance(Tables 2-3), the timing of peak eosinophil count emerged as an important factor. After excluding two patients with both PFS and OS below the five-week median cutoff and another two patients on concurrent chemotherapy and immunotherapy, landmark analysis showed that peak eosinophil timing ≤5 weeks was significantly associated with worsened PFS (7.9 vs 3.5 months, HR 3.6; p = 0.01; Benjamini-Hochberg-adjusted p = 0.02) (Figure 5, Table 4). Multivariate analysis further reinforced this finding (HR 7; p = 0.007) (Table 5). In contrast, OS was not significantly affected by time to eosinophil peak in either univariate (HR 2.3; p = 0.1) (Figure 6, Table 6) or multivariate analyses (HR 2.6; p = 0.1) (Table 7).

**Table 2:** Univariate analysis of PFS Univariate analysis of the clinical parameters for progression-free survival (PFS) in patients with metastatic pLELC receiving immunotherapy. None of the assessed clinical parameters reached statistical significance for association with PFS in this cohort. PDL-1: programmed cell death ligand 1; 1L: first-line treatment; 2L: second-line or subsequent treatment; CI: confidence interval; ENR: eosinophil-to-neutrophil ratio; NLR: neutrophil-to-lymphocyte ratio; CRT: chemoradiotherapy; PFS: progression-free survival

Variable	Median PFS (months)	Median PFS, 95% lower CI (months)	Median PFS, 95% upper CI (months)	Hazard ratio	p-value
Non-smoker	3.9	3.3	-	0.7	0.5
Smoker	5.9	2.4	9.6		
ECOG 1	7.7	2.7	9.6	1.25	0.6
ECOG 2	3.5	1	5.9		
PD-L1 < 50%	3.7	1	7.7	0.5	0.1
PD-L1 ≥ 50%	7.9	3.3	10.5		
1L, PD-L1 ≥1%	8.8	5.9	-	0.5	0.6
1L, PD-L1 <1%	11	-	-		
2L, PD-L1 ≥1%	3.5	1	7.7	1.4	0.7
2L, PD-L1 <1%	3.5	1.9	-		
1L	9.6	5.9	11	2.4	0.06
2L	3.4	1.9	4.8		
2L (Atezolizumab)	5.6	1.9	-	1.9	0.2
2L (Nivolumab)	2.7	1	4.8		
De Novo metastatic	4.6	1.9	9.6	0.8	0.5
Recurrence	4.8	1.9	7.9		
Eosinophil < 0.3	3.5	1.9	7.7	0.7	0.3
Eosinophil ≥ 0.3	7.9	2.7	10.5		
Eosinophil < 5%	4.8	1.9	9.6	0.9	0.9
Eosinophil ≥ 5%	7.7	2.7	-		
No prior CRT	4.8	2.7	7.9	0.5	0.2
Prior CRT	8.7	0.4	-		

**Table 3:** Univariate analysis of OS Univariate analysis of the clinical parameters for overall survival (OS) in patients with metastatic pLELC receiving immunotherapy. None of the assessed clinical parameters reached statistical significance for association with OS in this cohort. PD-L1: programmed cell death ligand 1; 1L: first-line treatment; 2L: second-line or subsequent treatment; CI: confidence interval; ENR: eosinophil-to-neutrophil ratio; NLR: neutrophil-to-lymphocyte ratio; CRT: chemoradiotherapy; OS: overall survival

Variable	Median OS (months)	Median OS, 95% lower CI (months)	Median OS, 95% upper CI (months)	Hazard ratio	p-value
Non-smoker	5.3	3.6	-	0.3	0.09
Smoker	22.4	8.2	26.6		
ECOG 1	22.2	5.3	26.6	1.4	0.5
ECOG 2	8	1	-		
PD-L1 < 50%	15.2	1	-	0.6	0.4
PD-L1 ≥ 50%	26.3	3.6	-		
1L, PD-L1 ≥1%	26.3	-	-	3.3	0.4
1L, PD-L1 <1%	22.4	3.6	-		
2L, PD-L1 ≥1%	8	1	-	0.8	0.7
2L, PD-L1 <1%	8.2	3.5	-		
1L	26.3	3.6	-	1.9	0.3
2L	8.2	3.5	23.6		
2L (Atezolizumab)	22.2	3.5	-	2.8	0.1
2L (Nivolumab)	8	1	-		
De Novo metastatic	22.4	4.8	26.3	1.7	0.3
Recurrence	8.1	3.4	-		
Eosinophil < 0.3	22.2	3.4	26.3	1.4	0.5
Eosinophil ≥ 0.3	23.6	5.3	-		
Eosinophil < 5%	22.2	3.5	26.6	1	1
Eosinophil ≥ 5%	23.6	5.3	-		
No prior CRT	22.4	5.3	26.3	1.6	0.4
Prior CRT	8	0.4	26.6		

**Table 4:** Univariate analysis of PFS (landmark cohort, n=22) This table presents univariate analyses assessing associations between haematological markers (eosinophil-to-neutrophil ratio (ENR), neutrophil-to-lymphocyte ratio (NLR), and timing of eosinophil peak) and progression-free survival (PFS) in the landmark cohort. Median PFS, 95% confidence intervals, hazard ratios, and both raw and Benjamini-Hochberg-adjusted p-values are displayed. The Benjamini-Hochberg false discovery rate correction was applied to account for multiple testing. Comparisons use the Kaplan-Meier method, with hazard ratios from Cox proportional hazards models. Lower ENR and early eosinophil peaks indicate poorer prognosis. Two patients with PFS below the five-week median cutoff and another two patients on concurrent chemotherapy and immunotherapy were excluded (n=22). CI: confidence interval; ENR: eosinophil-to-neutrophil ratio; NLR: neutrophil-to-lymphocyte ratio; PFS: progression-free survival

Variable	Median PFS (months)	Median PFS, 95% lower CI (months)	Median PFS, 95% upper CI (months)	Hazard ratio	p-value	Benjamini-Hochberg adjusted p-value
ENR ≥ 0.054	7.7	2.7	10.5	2.8	0.045	0.045
ENR < 0.054	3.4	1.2	7.7			
NLR < 2.64	7.9	3.5	10.5	4.3	0.01	0.02
NLR ≥ 2.64	3.3	1.9	-			
Peak eosinophil >5 weeks	7.9	3.3	10.5	3.6	0.01	0.02
Peak eosinophil ≤5 weeks	3.5	1.2	4.8			

**Table 5:** Multivariate analysis of PFS (landmark cohort, n=22) This table details the results of multivariate Cox proportional hazards analysis examining the independent predictors of progression-free survival (PFS) in the landmark cohort. Variables with univariate significance (duration to eosinophil peak ≤5 weeks and peak ENR 0.054) were included. Hazard ratios and p-values indicate that both earlier eosinophil peak and lower ENR are independently associated with worse PFS. Two patients with PFS below the five-week median cutoff and another two patients on concurrent chemotherapy and immunotherapy were excluded (n=22). ENR: eosinophil-to-neutrophil ratio; NLR: neutrophil-to-lymphocyte ratio; PFS: progression-free survival

Predictor	p-value	HR	95% Confidence interval
Duration to peak ≤ 5 weeks	0.007	7	1.7-29.2
ENR < 0.054	0.04	2.9	1.04-8.1

**Table 6:** Univariate analysis of the OS (landmark cohort, n=22) This table displays univariate analyses of the overall survival (OS) based on ENR, NLR, and eosinophil peak timing in the landmark cohort. The table shows median OS, 95% confidence intervals, hazard ratios, and p-values, applying the Kaplan-Meier and Cox model approaches. No marker demonstrated significant prognostic value for OS in these analyses. Two patients with OS below the five-week median cutoff and another two patients on concurrent chemotherapy and immunotherapy were excluded (n=22). CI: confidence interval; ENR: eosinophil-to-neutrophil ratio; NLR: neutrophil-to-lymphocyte ratio; OS: overall survival

Variable	Median OS (months)	Median OS, 95% lower CI (months)	Median OS, 95% upper CI (months)	Hazard ratio	p-value
ENR ≥ 0.054	23.6	8	-	1.5	0.4
ENR < 0.054	13.5	1.5	-		
NLR < 2.64	23.6	8	-	1.4	0.6
NLR ≥ 2.64	10.7	3.4	-		
Peak eosinophil >5 weeks	23.6	10.7	26.6	2.3	0.1
Peak eosinophil ≤5 weeks	6.7	1.5	-		

**Table 7:** Multivariate analysis of the OS (landmark cohort, n=22) This table summarises the multivariate analysis findings for the overall survival (OS) in the landmark cohort. Included variables are time to eosinophil peak and ENR, with hazard ratios and p-values reported. Neither factor reached statistical significance for OS. Two patients with OS below the five-week median cutoff and another two patients on concurrent chemotherapy and immunotherapy were excluded (n=22). ENR: eosinophil-to-neutrophil ratio; NLR: neutrophil-to-lymphocyte ratio; OS: overall survival

Predictor	p-value	HR	95% Confidence interval
Duration to peak ≤ 5 weeks	0.1	2.6	0.8-8.3
ENR < 0.054	0.8	1.1	0.4-3.6

**Figure 7:**
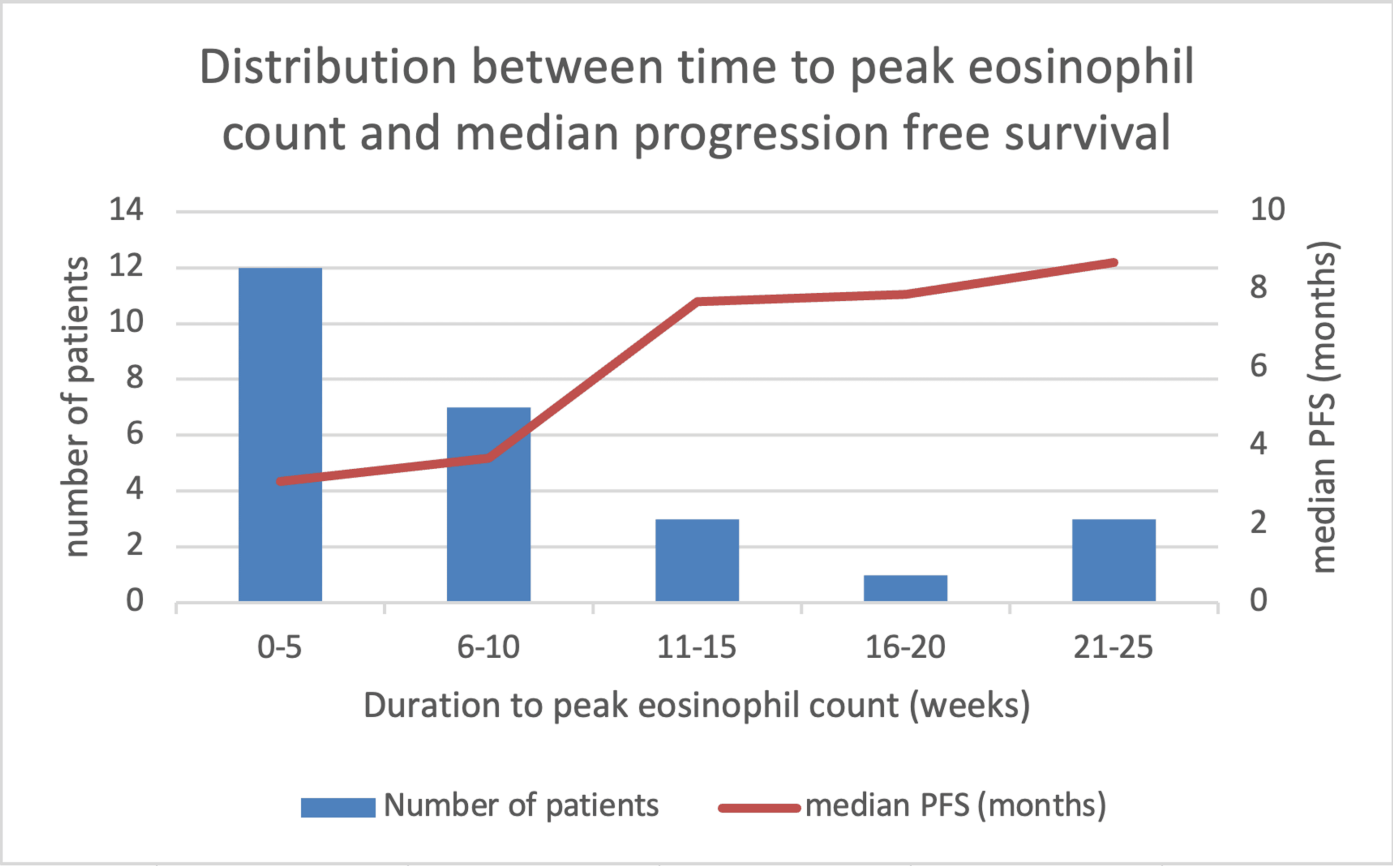
Distribution between time to peak eosinophil count and median PFS The relationship between duration to peak eosinophil count and median progression-free survival (PFS) in patients with metastatic pLELC treated with immunotherapy is shown in Figure 7. The bar chart depicts the number of patients stratified by intervals of time to eosinophil peak, while the overlaid line shows the median PFS for each interval. A right-skewed distribution was observed, with most patients reaching peak within 10 weeks. Delayed time to eosinophil peak was associated with a stepwise improvement in median PFS, highlighting the prognostic significance of eosinophil kinetics. PFS: progression-free survival

The distribution of the duration to eosinophil peak in our cohort remains right-skewed, with the majority of patients experiencing their peak within the first 10 weeks (median: five weeks, IQR: 2-10 weeks), while a minority had peaks as late as 25 weeks. Median PFS increased stepwise with delayed peaks, from 3.1 months (0-5 weeks) to 8.7 months (21-25 weeks) (Table 8). These findings indicate that, although early eosinophil peaks are common, a delayed eosinophil response is associated with progressively improved prognosis. This parallel right-skewed distribution for both the duration to eosinophil peak and PFS further supports the conclusion that a delayed eosinophil response is associated with improved clinical outcomes in this cohort (Figure 7).

**Table 8:** Distribution between the time to peak eosinophil count and median PFS This table displays the relationship between the duration (in weeks) to peak eosinophil count after immunotherapy initiation and median progression-free survival (PFS) among patients. For each interval, the number of patients and the corresponding median PFS are shown. This table underscores the correlation between delayed eosinophil peak and improved PFS in the cohort. PFS: progression-free survival

Duration to Peak Eosinophil (weeks)	Number of Patients	Median PFS (months)
0–5	12	3.1
6–10	7	3.7
11–15	3	7.7
16–20	1	7.9
21-25	3	8.7

The study evaluated post-treatment ENR and NLR for survival outcomes, using median cutoffs (ENR: 0.054, NLR: 2.64). While both lower ENR and higher NLR were associated with shorter PFS in univariate analyses after being adjusted for multiple comparisons using the Benjamini-Hochberg FDR method, only ENR 0.054 retained independent significance for poorer PFS within the landmark multivariate analysis (HR 2.9; p = 0.04) (Table 5). Neither ENR nor NLR was significantly associated with OS in any analytic model (Tables 6-7). Sensitivity analyses consistently confirmed the prognostic value of early eosinophil peak timing as the most robust predictor of shorter PFS, while the prognostic value of ENR appears more context-dependent and may be influenced by concurrent chemoimmunotherapy or sample size limitations. These findings further emphasise the preliminary, hypothesis-generating nature of ENR as a prognostic biomarker in this setting, highlighting the need for validation in larger cohorts (Tables 9-10).

**Table 9:** Sensitivity analysis for the multivariate analysis of PFS for full cohort (including chemoimmunotherapy, n=26) This table summarises the results of multivariate Cox regression analysis for progression-free survival (PFS) in all pulmonary lymphoepithelioma-like carcinoma (pLELC) patients who received immunotherapy, including those treated with concurrent chemoimmunotherapy. The table evaluates three candidate prognostic biomarkers: eosinophil-to-neutrophil ratio (ENR  <0.054 vs ≥ 0.054), duration to peak eosinophil count following treatment initiation (≤5 weeks vs >5 weeks), and neutrophil-to-lymphocyte ratio (NLR ≥ 2.64 vs <2.64). For each variable, the hazard ratio (HR), p-value, and 95% confidence interval are provided, indicating their independent impact on PFS. A significant association was observed for shorter duration to peak eosinophil (≤5 weeks), suggesting early peak is associated with poorer PFS in this cohort. ENR: eosinophil-to-neutrophil ratio; NLR: neutrophil-to-lymphocyte ratio; PFS: progression-free survival

Predictor	p-value	HR	95% Confidence interval
ENR < 0.054	0.3	2.0	0.6-7.2
Duration to peak ≤ 5 weeks	0.002	5.2	1.9-14.8
NLR ≥2.64	0.4	1.8	0.5-6.7

**Table 10:** Sensitivity analysis for the multivariate analysis of OS of full cohort (including chemoimmunotherapy, n=26) This table presents a multivariate Cox regression analysis assessing overall survival (OS) in patients with pulmonary lymphoepithelioma-like carcinoma (pLELC) treated with immunotherapy, including those who received concurrent chemoimmunotherapy. The model evaluates the independent prognostic impact of (1) time to peak eosinophil count following treatment initiation (≤5 weeks vs >5 weeks), (2) neutrophil-to-lymphocyte ratio (NLR ≥ 2.64 vs <2.64), and (3) eosinophil-to-neutrophil ratio (ENR  <0.054 vs ≥ 0.054). Hazard ratios (HR), p-values, and 95% confidence intervals (CI) are reported for each variable. Although the time to eosinophil peak reached statistical significance in this analysis, its prognostic value was not maintained in subsequent landmark analyses. OS: overall survival

Predictor	p-value	HR	95% Confidence interval
Duration to peak ≤ 5 weeks	0.006	11.7	2-68.1
NLR ≥2.64	0.3	1.6	0.6-4.5
ENR < 0.054	0.2	0.4	0.07-1.9

## Discussion

Our study is the first study to provide critical insights into the prognostic role of eosinophil dynamics for metastatic pLELC in response to IO, paralleling the findings with those observed in NSCLC [23-25]. The five-week cutoff for defining early versus delayed eosinophil peak was chosen empirically, as the median time to eosinophil peak in our cohort was five weeks (IQR: 2-10 weeks; Table 8, Figure 7). This data-driven threshold aligns with the findings in prior NSCLC studies. Okauchi et al. [24] suggested that the median time to the maximum eosinophil percentage was five weeks in patients with disease control and only two weeks in patients with progressive disease, concluding that monitoring peripheral eosinophils for up to five weeks - especially with respect to the timing and magnitude of the peak - can provide valuable prognostic information regarding immunotherapy efficacy. Takeuchi et al. [25] similarly demonstrated that an increased relative eosinophil count at four weeks (closely matching our five-week cutoff) independently predicted better disease control and overall survival. It is important to emphasise that our cutoff lacks independent biological or external validation. The use of a cohort-specific, retrospective threshold may introduce a risk of overfitting; thus, our findings should be regarded as preliminary and hypothesis-generating. We believe that this work provides a useful basis for further research, including prospective studies with pre-specified or externally validated cut-offs, to clarify the prognostic value of eosinophil kinetics in pLELC.

In recent years, there has been a growing emphasis on employing standard laboratory tests as clinical tools to predict the outcomes and responses of IO. These factors have generally been studied in small patient cohorts and have not been included in multivariate analyses [23-30]. With further validation, we recommend regular monitoring of eosinophil dynamics through complete blood counts every two to three weeks, which are routinely performed before each cycle of systemic therapy. This approach allows the early identification of patients who experience eosinophil peaks within five weeks of treatment initiation - a subgroup shown to have significantly poorer PFS in both univariate and multivariate analyses. Such patients may require intensified clinical monitoring and customised education to prepare for potential adverse outcomes. Our findings underscore the prognostic significance of the timing of eosinophil peak emergence, rather than focusing solely on absolute counts or percentages.

Preclinical studies indicate that eosinophils play a crucial role in enhancing anti-tumour responses by guiding T cells into the tumour, leading to tumour eradication and increased survival rates. Eosinophils achieve this through chemoattractants and degranulation, as well as altering the tumour microenvironment, which includes macrophage polarisation and vasculature normalization [31]. The presence of peripheral and tumour-associated eosinophilia is linked to better cancer prognosis, facilitated by the Th2 response and IL-5 production. Eosinophils also aid in the activation of dendritic cells and recruitment of T cells, suggesting a pivotal role in tumour surveillance and rejection to achieve a better disease control in response to IO [31-35].

This study is limited by its retrospective design, the rarity of pLELC, and a small sample size (n=26), which together introduce risks of selection and information bias, and restrict statistical power and generalisability. Selection bias was minimised by including all eligible patients, although information bias inherent to retrospective data collection remains a concern. To minimise the risk of false-positive findings due to multiple comparisons, we applied the Benjamini-Hochberg FDR correction to our univariate analyses. The associations remained statistically significant after this adjustment, underscoring the robustness of our findings. Analytical strategies, such as landmark analysis and the exclusion of patients treated with CIO, were employed to address immortal time bias and confounding - particularly their potential impact on neutrophil counts. However, these measures may have resulted in underrepresentation of patients with aggressive disease, further reducing the size and diversity of the analysis cohort. We observed right-skewed distributions for both PFS and time to peak eosinophil, with most patients experiencing short durations and a minority experiencing much longer ones; hence, medians were reported for all descriptive statistics. Sensitivity analyses confirmed the robustness of our findings to outliers and analytic decisions. Despite efforts to ensure robustness with sensitivity analyses, the findings should be interpreted with caution. Larger, prospective studies are needed to validate the prognostic significance of eosinophil kinetics.

The analysis of ENR and NLR was limited by a small sample size (n=22) and possible confounding factors. The NLR cut-off in our analysis was informed by recent literature in lung cancer, which reported an NLR threshold of 2.6 for mortality prediction [36]. For ENR, no universal cut-off exists; our threshold was determined pragmatically from cohort distribution and supported by prior exploratory studies [26]. We acknowledge that these values require further validation in larger, prospective studies and that consensus on optimal cut-offs is still evolving. Similar to the broader associations observed by Tanizaki et al. [23] in NSCLC, our study found that a lower ENR was associated with worsened PFS in patients with pLELC receiving IO, but no significant association with OS was observed in either univariate or multivariate analyses.

The small sample size and single-cohort, retrospective study design introduce limitations regarding statistical power and potential for overfitting. As such, these associations should be interpreted as preliminary observations rather than definitive conclusions. Our results align with and extend previous exploratory work in NSCLC and related malignancies, but the applicability of these cut-offs and markers in broader clinical settings remains unknown. The potential utility of eosinophil kinetics and related haematological markers for risk stratification warrants further investigation in larger, prospectively designed, and multi-institutional cohorts, with independent validation of cut-offs before clinical implementation.

## Conclusions

In conclusion, our study provides preliminary evidence suggesting that early eosinophil peak and low ENR may be associated with poorer PFS in metastatic pLELC patients undergoing immunotherapy, with the potential to enhance patient management and resource allocation. These hypothesis-generating findings suggest that eosinophil kinetics may not only inform prognosis but could also be leveraged to optimise treatment, warranting further prospective investigation. Future studies with larger, multi-institutional patient cohorts and prospective data collection are necessary to validate these findings and enhance their clinical applicability.
